# Supplementary material for: Content-rich biological network constructed by mining PubMed abstracts
Source: BMC Bioinformatics. 2004 Oct 8;5:147. doi: 10.1186/1471-2105-5-147 (PMC528731; doi:10.1186/1471-2105-5-147)
Supplement: Additional File 2 — The original results of the above study (non-essential files are deleted to keep the file size under the limit set by BMC bioinformatics). [file 1471-2105-5-147-S2.bz2 › chilibotAdditionalFile2/dip10/448ID9115216E10455/html/CCR1_CCL2.html]

 


 **CCR1** and **CCL2** 
  
Found 155 abstracts in PubMed, retrieved 10.  
 

 What does Google say? 
 PDF only 
| .edu only 

---

**Interactive relationship** (e.g. stimulation, inhibition, etc)

**Neutral relationship**- Functional studies and binding experiments using biotinylated monocyte chemoattractant protein MCP 1  [ **CCL2** ]   **CCL2**  indicate that  **CCL2**  could be a candidate chemokine ligand for glial L CCR  [ **CCR1** ] .  Ref: 12555200 Glia, 2003
- Expression of L CCR  [ **CCR1** ]  in HEK 293 cells reveals functional responses to  **CCL2** , CCL5, CCL7, and CCL8.  Ref: 12885941 J Leukoc Biol, 2003
- Biotinylated  **CCL2**  binds to L CCR  [ **CCR1** ]  expressing cells, and transfection experiments with an L CCR  [ **CCR1** ]  green fluorescent protein fusion protein showed L CCR  [ **CCR1** ]  expression in the membranes of recombinant HEK 293 cells.  Ref: 12885941 J Leukoc Biol, 2003
- The G protein coupled receptor CCR  [ **CCR1** ]  2B is probably the most important MCP 1  [ **CCL2** ]  receptor in vivo, and loss of MCP 1  [ **CCL2** ]  effector function alone is sufficient to impair monocytic trafficking in inflammation models.  Ref: 12556201 Expert Opin Ther Targets, 2003
- The present findings suggest that MCP 1  [ **CCL2** ] , RANTES, and IP 10 may participate in the pathogenesis of mercuric chloride induced tubulointerstitial fibrosis with mononuclear cell infiltration, via CCR2,  **CCR1**  or CCR5, and CXCR3, respectively.  Ref: 12834626 Exp Mol Pathol, 2003

**Non-interactive relationship** (e.g. studied together, co-existance, homology, etc.)

- In parallel,  **CCR1**  and CCR5 expression is down regulated and CXCL8,  **CCL2** , CCL3 and CCL5 production is up regulated.  Ref: 12548563 Eur J Immunol, 2003
- Several selective small molecular weight CCR  [ **CCR1** ]  2B antagonists and MCP 1  [ **CCL2** ]  blocking antibodies have been described.  Ref: 12556201 Expert Opin Ther Targets, 2003
- We studied polymorphisms in the genes for monocyte chemoattractant protein 1  [ **CCL2** ]  MCP 1  [ **CCL2** ]  and CC chemokine receptor CCR  [ **CCR1** ]  2 in 171 Parkinson s disease PD patients and 340 controls.  Ref: 12889089 Mov Disord, 2003
